# Description of *Bryantinus* gen. n. from Sarawak, and New Distributional Records for *Cerochusa cilioceps* in Thailand (Coleoptera: Staphylinidae: Pselaphinae)

**DOI:** 10.1371/journal.pone.0113474

**Published:** 2014-11-19

**Authors:** Zi-Wei Yin, Li-Zhen Li

**Affiliations:** Department of Biology, College of Life and Environmental Sciences, Shanghai Normal University, Shanghai, P. R. China; University of Idaho, United States of America

## Abstract

A new genus and species of the subtribe Batrisina from western Sarawak, *Bryantinus matangus* **gen. et sp. n.**, is described, illustrated, and compared with related taxa. In addition, examination of a small series of batrisine material from Thailand revealed a new country record for *Cerochusa cilioceps* Yin & Nomura, which was previously known only from the island of Hainan in southern China.

## Introduction

The ant-loving beetles, or the subfamily Pselaphinae Latreille, is one of the megadiverse groups of the rove beetle family Staphylinidae Latreille. A total of 9,801 species placed in 1,252 genera (including 19 extinct genera and 41 extinct species) [A. F. Newton, unpublished database, 1st October, 2014], grouped in six or seven supertribes [1], [2], are known worldwide. Members can be found in all major zoogeographic regions, but are most diverse in tropics [1], [3]. The Palaearctic fauna is relatively well-studied at both the generic and species levels, and a catalog has been published [4]. The genera of North America and Mexico are largely known [5], [6], [7], those of the Neotropical region were treated in Park's works [8], [9], [10], and temperate South America was covered by Jeannel [11]. In the Oriental region, the pselaphine fauna is still poorly studied in most areas, and many biodiversity hotspots have never been thoroughly sampled. Recently, Nomura and his collaborators published a series of species checklists covering China (Yunnan), Malaysia, Singapore, Borneo, Thailand, and Vietnam, as the first step toward modern taxonomic studies of the pselaphine fauna in these areas [12], [13], [14], [15], [16], [17]. As to Batrisitae, only a small number of genera, e.g. *Mnia* Raffray, *Cratna* Raffray, *Sathytes* Westwood, etc. were revised by recent workers [18], [19], [20], while many others still await such treatments. One case indicating the chaotic taxonomic status of Asian pselaphines is that Nomura & Idris listed 109 batrisine species from Malaysia and Singapore [13], among these, many have been placed in the genus *Batrisodes* since Raffray's time [21], [22]. It is possible that none of these species truly belongs to the largely Holarctic genus *Batrisodes*, and may be moved to other genera in the future, or represent undescribed genera, as partially illustrated by Kurbatov [23].

Our recent studies on the Southeast Asian Batrisitae have documented a number of new taxa [24], [25], [26], but the gap of the knowledge of the existing richness of this diverse group remains large. In the course of our on-going study of Asian Pselaphinae currently, we are focusing on descriptions of the large loan of material from the Muséum d'histoire naturelle de la Ville de Genève. From this several undescribed genera and numerous undescribed species were noticed. In this paper, we describe a new genus and species of the tribe Batrisini from western Sarawak that is placed near *Cerochusa* Yin & Nomura and *Ceroderma* Raffray, and provide new distributional data for the previously described *Cerochusa cilioceps* Yin & Nomura known from Hainan Island of South China.

## Materials and Methods

The material treated in this study is housed in the Muséum d'histoire naturelle, Geneva (**MHNG**) and the Insect Collection of the Shanghai Normal University, Shanghai (**SNUC**).

The collection data of the material are quoted verbatim. A slash (/) is used to separate different labels. Depository of type material of respective species is indicated by their type label.

The terminology follows Chandler, 2001 [1], except we use ‘ventrite’ instead of ‘sternite’ when describing meso- and metathoracic structures. The terms ‘tergite’ and ‘sternite’ are used when referring to abdominal segments [27].

The following abbreviations are applied: **AL**–length of the abdomen along the midline; **AW**–maximum width of the abdomen; **EL**–length of the elytra along the sutural line; **EW**–maximum width of the elytra; **HL**–length of the head from the anterior clypeal margin to the occipital constriction; **HW**–width of the head across eyes; **PL**–length of the pronotum along the midline; **PW**–maximum width of the pronotum. Length of the body is a combination of HL+PL+EL+AL.

### Nomenclatural acts

The electronic edition of this article conforms to the requirements of the amended International Code of Zoological Nomenclature, and hence the new names contained herein are available under that Code from the electronic edition of this article. This published work and the nomenclatural acts it contains have been registered in ZooBank, the online registration system for the ICZN. The ZooBank LSIDs (Life Science Identifiers) can be resolved and the associated information viewed through any standard web browser by appending the LSID to the prefix “http://zoobank.org/”. The LSID for this publication is: urn:lsid:zoobank.org:pub:6BD08956-763D-4033-9399-184C5933C87F. The electronic edition of this work was published in a journal with an ISSN, and has been archived and is available from the following digital repositories: PubMed Central, LOCKSS.

## Results and Discussion

### *Bryantinus* Yin and Li, new genus

urn:lsid:zoobank.org:act:6AEB3CCA-71D3-48C6-9216-0238EE0A4E31

#### Type species

*Bryantinus matangus*, new species (here designated)

#### Diagnosis

Head triangular; lacking frontal rostrum; lacking frontal fovea; antennomeres XI robust and conical. Pronotum transversely oval, lateral margins angulate and incised posterior to level of lateral antebasal foveae; lacking median antebasal fovea; lacking antebasal spines. Elytra lacking basal foveae, discal striae shallow and short. Abdominal tergites IV–VII with thin marginal carinae; tergite IV longest; discal carinae absent.

#### Description

**Figure 1:**
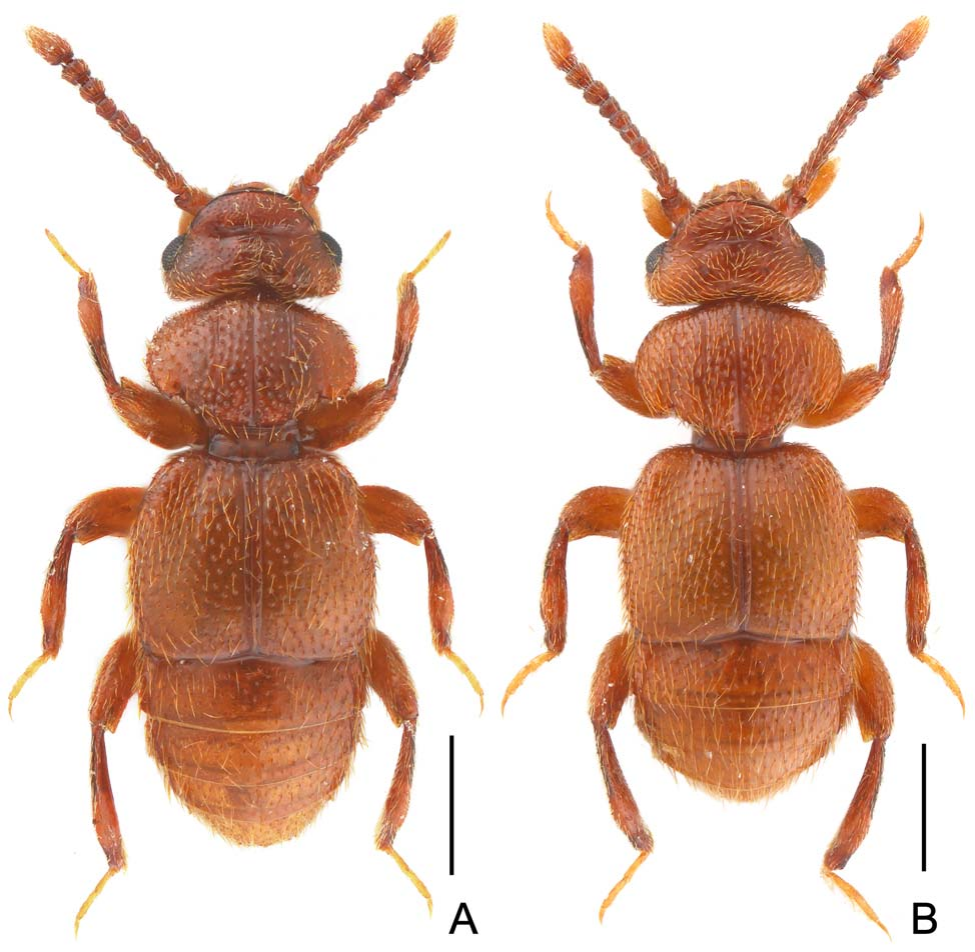
Habitus of *Bryantinus matangus*. (A) Male. (B) Female. Scales: 0.5 mm.

**Figure 2:**
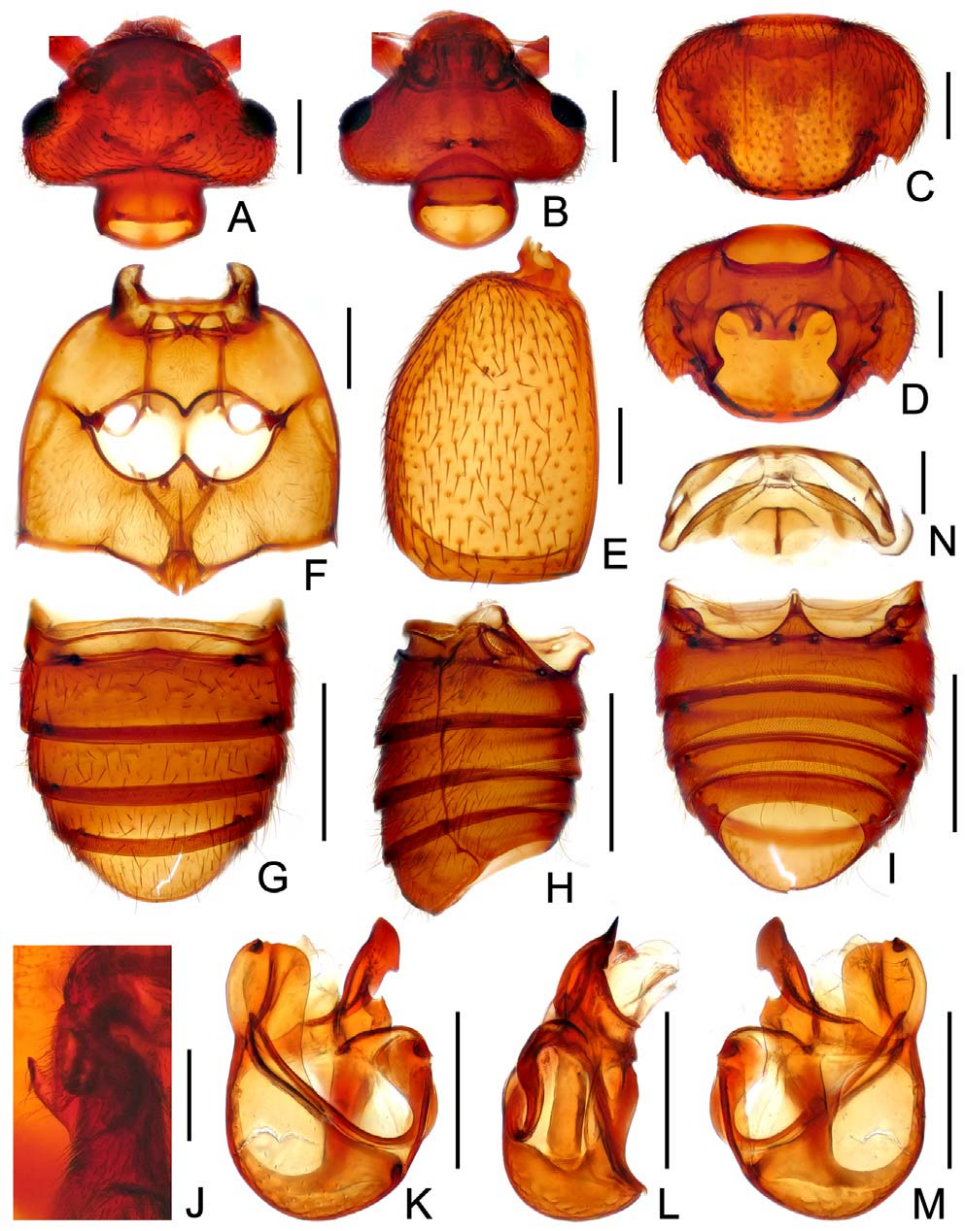
Diagnostic features of *Bryantinus matangus*. (A) Head, in dorsal view. (B) Same, in ventral. (C) Pronotum. (D) Prosternite. (E) Left elytron. (F) Meso- and metaventrite. (G) Abdomen, in dorsal view. (H) Same, in lateral view. (I) Same, in ventral view. (J) Male metatrochanter. (K) Aedeagus, in dorsal view. (L) Same, in lateral view. (M) Same, in ventral view. (N) Female genital complex, in dorsal view. Scales: A–F, K–M = 0.2 mm; G–I = 0.5 mm; J, N = 0.1 mm.

Body flat and broad (Fig. 1); length 2.26–2.30 mm. Head transversely triangular (Figs. 2A, B), lacking frontal rostrum, antennal tubercles weak; lacking frontal fovea; vertex slightly convex medially, vertexal foveae small, connected by shallow, barely noticeable U-shaped sulcus; postocular margins rounded; posterior margin moderately impressed medially; with eleven antennomeres, clubs weakly indicated by apical three antennomeres, antennomeres XI robust and conical; ocular-mandibular carinae well-defined; eyes oval, with many small facets; maxillary palpi with palpomeres III small and triangular, IV gradually narrowed in basal half; small gular foveae close.

Pronotum (Fig. 2C) transversely oval; with lateral antebasal foveae, lacking median antebasal foveae; with distinct median sulcus, lacking lateral longitudinal sulci, thin discal carinae extending anteriorly from middle to near apical margin; lateral spines distinct, lacking antebasal spines; lateral margins incised posterior to level of lateral antebasal foveae; disc only slightly convex; lacking basolateral foveae; sinuate paranotal carinae distinct (Fig. 2D); lateral procoxal foveae small. Elytra (Fig. 2E) lacking basal foveae, discal striae shallow and short; sutural and marginal striae complete; lacking subhumeral foveae. Thorax (Fig. 2F) with small median and forked lateral mesoventral foveae; lateral mesocoxal foveae present; with small lateral metaventral foveae; posterior margin narrowly notched medially. Legs with thickened femora, tarsomeres II and III subequal in length.

Abdomen (Figs. 2G–I) with tergite IV (first visible tergite) longer than tergite V; lateral margins of tergite IV–VII edged by thin marginal carinae, which extend entire tergal length; tergite IV with broad basal impression and one pair of lateral foveae; lacking discal carinae; tergites V–VII lacking basal impression, each with one pair of small lateral foveae. Sternite IV with small median and one pair of lateral foveae, lacking basal impression; sternites V–VII lacking foveae.

Male with vertex slightly modified, transverse sulcus slightly broader and deeper than that in female. Aedeagus with paramere fused to median lobe to form elongate ventral lobe; with semi-sclerotized dorsal lobe.

#### Comparative notes

The new genus is placed near *Cerochusa* and *Ceroderma*, and may be a sister taxon of the former. All of these taxa share a flat body form, a triangular head, a transverse pronotum, and placement of the male features on the head dorsum. *Bryantinus* shares with *Cerochusa* many character states and a similar foveal pattern, but the lateral pronotal longitudinal sulci and basal elytral foveae are lacking, while *Cerochusa* possesses a pair of lateral sulci on the pronotum, and has two basal foveae on each elytron. *Bryantinus* shares with *Ceroderma* the lack of discal carinae on tergite IV, but can be separated by the presence of basomedian and basolateral foveae on sternite IV; in contrast *Ceroderma* has three basal elytral foveae, and the basomedian and basolateral foveae of sternite IV are lacking.

#### Etymology

The new genus is named after Gilbert E. Bryant (1878–1965), who collected the material used in this work. The gender is masculine. The specific epithet refers to the type locality of the new species.

### *Bryantinus matangus* Yin and Li, new species

urn:lsid:zoobank.org:act:12CD7A88-A9D7-4DF8-936E-0964512DE132.

#### Type material (1 ♂, 5 ♀♀)

**Holotype: Malaysia:** ♂, labeled ‘Mt. Matang, W. Sarawak, G. E. Bryant, 26.I.14/G. Bryant Coll., B.M. 1926–86./HOLOTYPE [red], *Bryantinus matangus* sp. n., Yin & Li det., 2014, MHNG’. **Paratypes: Malaysia:** 1 ♀, same label data as holotype; 2 ♀♀, same locality and date, with additional data ‘No. 7. 14’; 2 ♀♀, same label data, except ‘1.II.14’, and ‘I.-II 1914’. Each paratype bears a type label as ‘PARATYPE [yellow], *Bryantinus matangus* sp. n., Yin & Li det., 2014, MHNG’.

#### Diagnosis

Head triangular; vertex convex medially, shallow U-shaped sulcus connecting small vertexal foveae; frons separated from clypeus by round anterior margin; postocular margins rounded; pronotal lateral margins incised posterior to level of lateral antebasal foveae; elytra barely constricted at base, lacking basal foveae.

#### Description

Male (Fig. 1A). BL 2.29 mm. Body reddish brown, tarsi lighter in color; most part of dorsal surface densely hairy.

Head distinctly transverse, HL 0.41 mm, HW 0.66 mm; vertex slightly convex, foveae below level of posterior margins of eyes, indistinct longitudinal impressions developed from foveae anteriorly toward broad, deeper transverse sulcus; eyes relatively small, each composed of about 110 small facets; antennal clubs weakly indicated by apical three slightly enlarged antennomeres. Pronotum nearly oval, transverse, PL 0.51 mm, PW 0.77 mm; lateral margins rounded, with dentate posterior margin. Elytra wider than long, EL 0.68 mm, EW 0.89 mm; lacking basal foveae. Metathoracic wings fully developed. Metatrochanters (Fig. 2J) each with truncate projection on ventral margin. Abdomen wider than long, AL 0.69 mm, AW 0.83 mm; tergites IV–VII margined by thin lateral carinae; sternites IV lacking basal sulcus, V–VII lacking ridge and fovea. Aedeagus (Figs. 2K–M) asymmetric, robust, length 0.63 mm; elongate ventral lobe with round apex, dorsal lobe flat, small part of apical portion curved mesally.

Female (Fig. 1B). Similar to male in general appearance, only distinguishable by presence of very thin, shallow transverse sulcus on frons, versus broader, slightly deeper one in male. Measurements: BL 2.26–2.30 mm, HL 0.43–0.44 mm, HW 0.70–0.71 mm, PL 0.52–0.53 mm, PW 0.81–0.84 mm, EL 0.68–0.71 mm, EW 0.89–0.93 mm, AL 0.60–0.65 mm, AW 0.86–0.87 mm.

#### Comparative notes

*Bryantinus matangus* can be readily separated from all other batrisines by the diagnostic features for the genus, combined with the male feature on the frons, which is probably a species-specific characteristic.

#### Distribution

East Malaysia: Sarawak.

### *Cerochusa cilioceps* Yin and Nomura

*Cerochusa cilioceps* Yin and Nomura, 2012 (in Yin, Nomura and Li, 2012: 68) [24].

#### Additional material examined (7 ♂♂)

2 ♂♂, labeled ‘THAILAND: Nan Prov., 1350–1800 m, Doi Phu Kha N. P., Schwendinger, 5.10.91’ (MHNG); 5 ♂♂, labeled ‘THAILAND: 1000–1250 m, Loei Prov., Phu Rua Nat. Park, Schwendinger, 5.9.92’ (MHNG, SNUC).

#### Distribution

**Figure 3:**
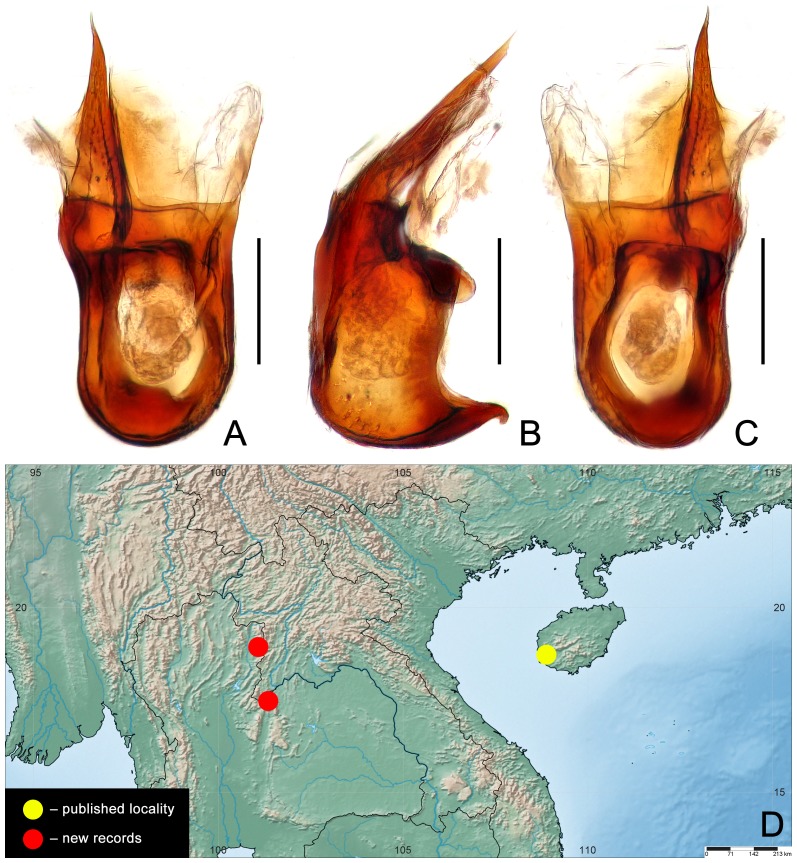
Aedeagus (A–C) and distribution (D) of *Cerochusa cilioceps* in Thailand. (A) Dorsal view. (B) Lateral view. (C) Ventral view. Scales: 0.1 mm.

South China: Hainan; Thailand: Nan, Loei (**new country record**) (Fig. 3D).

#### Comments

The form of the modifications on the head dorsum and the aedeagus (Figs. 3A–C) leave no doubt that the population from Thailand is conspecific with that from the type locality [24].
